# Public opinion concerning governments’ response to the COVID-19 pandemic

**DOI:** 10.1371/journal.pone.0260062

**Published:** 2022-03-02

**Authors:** Cathy W. S. Chen, Tsai-Hung Fan

**Affiliations:** 1 Department of Statistics, Feng Chia University, Taichung, Taiwan; 2 Graduate Institute of Statistics, National Central University, Taoyuan, Taiwan; Public Library of Science, UNITED KINGDOM

## Abstract

**Objectives:**

Governments around the world have implemented numerous policies in response to the COVID-19 pandemic. This research examines the political issues resulting in public opinion concerning their responses to the pandemic via an international perspective. The objectives of this study are to: (1) measure the association and determine whether differences in political support can be attributed to the presence of approval ratings during the pandemic, and to (2) identify exceptional cases based on statistical predictions.

**Methods:**

We collect information from several open-sourced surveys conducted between June and September 2020 of public sentiment concerning governments’ response toward COVID-19. The 11 countries in our sample account for over 50% of the world’s Gross Domestic Product (GDP). The study includes country-specific random effects to take into account the data’s clustered structure. We consider “political partisanship” and “pre-pandemic approval ratings in 2019” as two potential explanatory variables and employ a mix-effect regression for bounded responses via variable transformation and the wild bootstrap resampling method.

**Results:**

According to the wild bootstrap method, the mixed-effect regression explains 98% of the variation in approval ratings during the pandemic in September 2020. The findings reveal partisan polarization on COVID-19 policies in the U.S., with opposing supporters most likely to express negative sentiments toward the governing party.

**Conclusions:**

The evidence suggests that approval ratings during the pandemic correlate to differences in political support and pre-pandemic approval ratings, as measured by approval ratings from the views between governing coalition supporters and opponents.

## 1. Introduction

The COVID-19 pandemic has put governments worldwide under extreme pressure to react fast and decisively. Most of them have implemented numerous policies in response to the pandemic, but they vary substantially across countries. While the challenges appear to be similar for many governing parties, the political reactions differ markedly. The impact of the COVID-19 pandemic on human lives and political attitudes is clearly unquestionable.

Several contemporaneous studies target the impact of COVID-19 on political attitudes and behavior, such as [1, 2] comparing respondents’ political attitudes in 15 European countries and finding that public support for governing parties increases in response to lockdown policies. [3] conclude that approval of incumbent politicians falls as COVID-19 cases grow. [4] investigate the most important predictor variables influencing the satisfaction of citizens on their governments’ responses to the pandemic based on five covariates for analysis: the number of confirmed cases per million population, the number of deaths per million population, governments’ containment and health policies, their stringency policies, and their economic support policies. Their results reveal that people pay stronger attention to the “number” of government battles against COVID-19 rather than what policies a government may initiate.

Partisanship in many countries has an important influence on attitudes about a government’s policies. People who identify with the current ruling party are remarkably more satisfied with government policies than those who either support the opposition or identify with no political party. The papers mentioned above do not consider one crucial factor, political partisanship, when dealing with government approval issues regarding the COVID-19 pandemic. [5–7] pay attention to partisan differences in U.S. respondents’ views over the COVID-19 pandemic, but their results limit individual behavior and beliefs about the pandemic to be partisan.

This present study aims to measure the strength of relationship and association between variables and to determine whether differences in political support correlate to the presence of public opinion concerning governments’ responses amid the COVID-19 pandemic from an international perspective. We analyze a dataset of 11 advanced economies and discover the hidden factors on public opinion relating to governments’ responses to the pandemic. Each datapoint includes a survey result. The objectives of this study are to: (1) measure the association among pre-pandemic approval ratings, political partisanship, and pandemic approval ratings in 2020; to (2) investigate whether differences in political support and pre-pandemic approval ratings are due to the presence of approval ratings during the pandemic; and to (3) identify exceptional cases based on statistical predictions. The datasets come from public open-sourced surveys and are grouped by political partisanship. The study considers “political partisanship” and “pre-pandemic approval ratings in 2019” as two potential explanatory variables and incorporates a country-specific random effect as a mixed-effect regression for bounded responses via a variable transformation. One can avoid the shortcoming of multiple regression in this study by adding a random effect component in regression. Mixed-effects modeling allows us to examine the condition of interest while also taking into account variability within and across items simultaneously.

We adopt bootstrapping (or resampling) methods to overcome problems of unknown sampling distributions. The bootstrap, proposed initially by [8], approximates the unknown theoretical sampling distribution of the coefficient estimates by an empirical distribution obtained through a resampling process. This computer-based technique is powerful for presenting statistical inferences without requiring strong assumptions on the sample or the population. We employ wild bootstrap resampling methods by [9, 10] for making inferences as the sample does not conform to the assumptions of normality and homoskedasticity.

## 2. Data description

We collect the public open-sourced data concerning the “majorities of governing party supporters who say their country has dealt with the COVID-19 outbreak well” from the Global Attitudes Survey conducted in June through August 2020, which is available from the Pew Research Center [11]. However, [11] mainly focus on political division within the U.S. The purpose of this study is different from the literature mentioned above, as we examine the association and the effects of two potential explanatory variables on public opinion concerning governments’ responses amid the COVID-19 pandemic.

**Fig 1:**
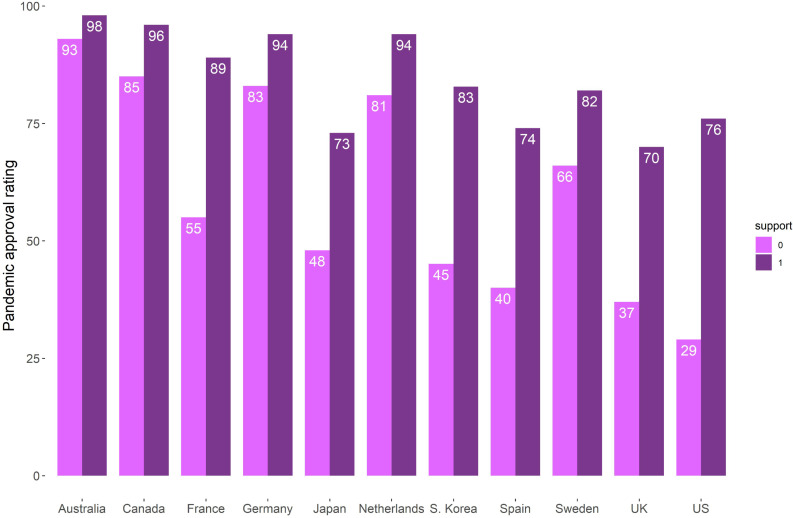
Approval ratings of respondents who “say their country has dealt with the COVID-19 outbreak well” grouped by governing party supporters (1) and non-supporters (0).

Fig 1 illustrates the approval ratings of respondents who “say their country has dealt with the COVID-19 outbreak well”, grouping them by governing party supporters and non-supporters. We observe among all countries surveyed that governing party supporters are more likely to say their government has done a good job than those who do not support the governing coalition.

The information of South Korea in Fig 1 is not included in the Pew Research Center data. The approval rating of South Korea is from [12] via a web survey from September 9–18, 2020, in which 82.81% of the respondents from the Democratic Party, the current party in charge of the country’s government, support the government’s approach to the pandemic, while 45.12% holding a “no party preference” are satisfied with the government’s response to the 2020 COVID-19 outbreak.

In our study there are 6 European countries (Germany, Netherlands, France, UK, Sweden, and Spain), 2 North American countries (the U.S. and Canada), one Oceania country (Australia), and 2 East Asia countries (Japan and South Korea). The pre-pandemic source is from [13], who present the economic attitudes among governing coalition supporters and opponents in 2019. Aside from the nine advanced economies in [13], our study further gathers information on Germany and South Korea. Therefore, we have 22 datapoints for 11 countries. The datapoint of Italy is not in this study since there was no information among governing coalition supporters and opponents in 2019. Nevertheless, these 11 countries in our sample account for over 50% of the world’s Gross Domestic Product (GDP) in 2020.

The information of Germany comes from the survey “in favor of Federal Chancellor remaining in office” in March 2019 [14]. In South Korea, with its presidential system, we follow the Gallup Korea Daily Opinion [15]. By political party in South Korea, 80% of Democratic Party supporters evaluated the president’s performance positively, while 20% of non-party supporters showed positive support in 2019.

**Fig 2:**
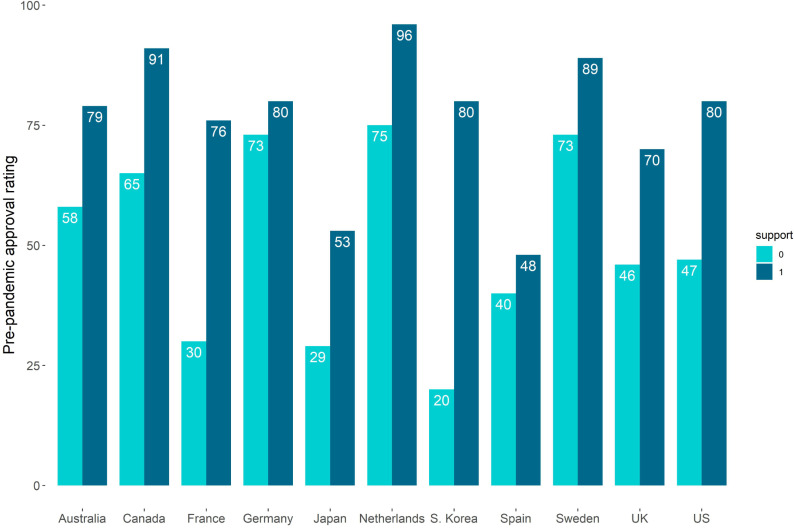
Pre-pandemic approval ratings in 2019 grouped by governing party supporters (1) and non-supporters (0).

Fig 2 exhibits pre-pandemic approval ratings, while Table 1 shows the summary approval ratings for the 11 countries pre-pandemic and amid the COVID-19 pandemic. The notation {Y_ij_} stands for an approval rating, like public opinion, concerning how the governing parties had done a good job dealing with the COVID-19 outbreak in September 2020, while {X_*i*1*j*_} stands for an approval rating for governing parties in 2019. Each country has two approval ratings based on governing party supporters (1) and non-supporters (0). We denote this variable as “Support.” We notice considerable heterogeneity in approval ratings during the pandemic in September 2020, as well as approval ratings for the pre-pandemic period in 2019.

**Table 1:** Descriptive statistics.

	Notation	Sample size	Min	Max	Mean	Std. dev.
Approval rating for COVID-19	{*Y*}	22	29	98	72.31	21.02
Pre-pandemic approval rating	{*X*_1_}	22	20	96	63.55	21.55
Political partisanship	{Support}	22	0	1		

## 3. Methodology

We first measure the association among all variables based on [16]. When the continuous variables of interest have extreme values, then in this case a more appropriate measure of a linear relationship is the Spearman rank correlation coefficient. Table 2 reports the Pearson correlation coefficient and Spearman rank correlation coefficient for continuous-continuous variables, {Y,*X*_1_}. All Pearson and Spearman rank correlation coefficients show only a slight difference. We provide a point-biserial correlation coefficient when dealing with continuous-nominal variables; i.e., {Y, support} and {X_1_, support}.

**Table 2:** Measure association of variables in the full sample and transformed data. A Pearson correlation coefficient is for {Y, X_1_} or {Resp, X_1_}, and Spearman rank correlation coefficient is in (). A point-biserial correlation coefficient appears in boldface.

		Y	*X*_1_	Support
Full sample	Y	1	0.7732 (0.7877)	**0.6703**
	*X*_1_	0.7732 (0.7877)	1	**0.6521**
	Support	**0.6703**	**0.6521**	1
Transformed data		Resp	*X*_1_	Support
	Resp	1	0.7507 (0.7732)	**0.5704**
	*X*_1_	0.7507 (0.7732)	1	**0.6175**
	Support	**0.5704**	**0.6175**	1

Since {Y_ij_}, the pandemic approval rating, is a bounded response variable, we employ a logistic transformation to handle the bounded response variables (see [4, 17]) so that its support becomes a real line. We specify that a regression model with random intercepts varies by country. To such an end, a useful reparameterization of the mixed-effect regression goes as follows:

$$Resp_{ij}=\alpha_{j}+\beta_{0}+\beta_{1}X_{1ij}+\beta_{2}Support_{ij}+\epsilon_{ij},\;i=1,2,\;j=1,\ldots ,k,$$ (1)

where $Resp_{ij}=\ln\left(\frac{Y_{ij}}{100-Y_{ij}}\right)\times 10$; *α_j_* is a country-specific random effect; {*Y*_*i*j_} is an approval rating of respondents who “say their country j has dealt with the COVID-19 outbreak well” in September 2020; and {*X*_1*i*j_} stands for approval rating for the governing parties of country j in 2019. We present governing party supporters and non-supporters through a subscript as group *i* and *Support*_*ij*_ = 1 if from the group of party supporters; otherwise, *Support*_*ij*_ = 0. The lower panel of Table 2 shows the association among three variables, {Resp, Y,*X*_1_}.

There are several popular bootstrap methods for regression, such as empirical bootstrap, residual bootstrap, and wild bootstrap [18]. The wild bootstrap developed in [10] helps overcome heteroskedasticity in the error term. We apply the wild bootstrap resampling method to the mixed-effect model in (1). The wild bootstrap calls for bootstrapping residuals from an “external” distribution. The following steps demonstrate how the wild bootstrap works in our analysis.

1. We fit a mixed-effect model in Eq (1). We save ($\hat{Resp_{ij}},\;e_{ij}$), which are the predicted values and the residual values, respectively.
2. We generate independent and identically distributed random variables V_ij_~*N*(0, 1), *i* = 1,2, *j* = 1,…,*k*.
3. We obtain the bootstrap sample $\left(Resp_{ij}^{*},X_{1ij},Support_{ij}\right)$ by:
$$Resp_{ij}^{*}=\hat{Resp_{ij}}+V_{ij}\times e_{ij},$$ (2)
4. We repeat Steps 2 and 3 B times and obtain B sets of fitted coefficients and R^2^.
5. We analyze the bootstrap distribution to estimate standard errors and confidence intervals for the parameters.

## 4. Results and discussion

**Fig 3:**
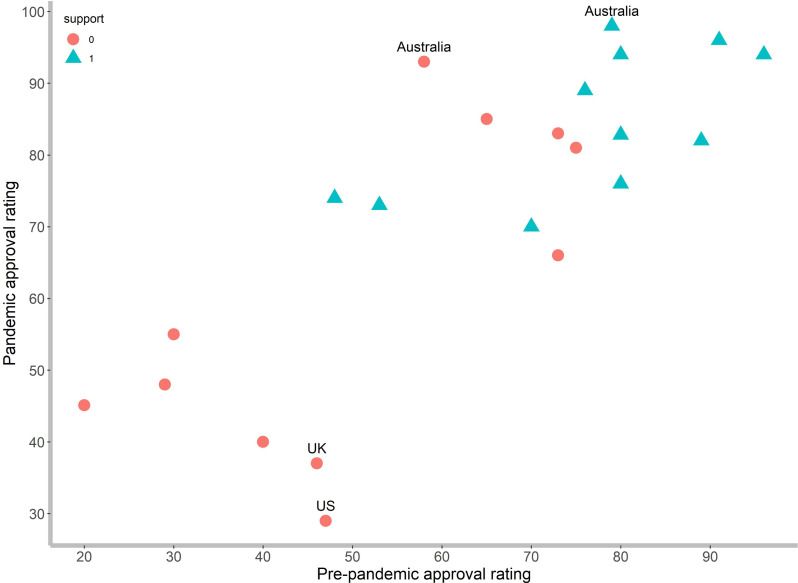
Scatter plot of pandemic approval ratings and pre-pandemic approval ratings, {Y_i_, X_1_}, by governing party supporters (1) and non-supporters (0).

We use a mixed-effect regression to investigate the relationship in which a country-specific random effect takes into account the data’s clustered structure. Grounded on 3000 bootstrap replicates, the mixed-effect regression explains 98% of the variation in “the government approval ratings amid the pandemic” (Table 3). The fixed-intercept estimate, $\hat{\beta_{0}}$ = -3.6584, represents the average intercept, and random intercepts allow each country to deviate from this average. This study shows that both “pre-pandemic approval ratings” and “political partisanship” variables significantly affect attitudes toward the pandemic. Fig 3 presents a scatter plot of pandemic approval ratings versus pre-pandemic approval ratings. However, the former is not our transformed response variable in Eq (1).

**Table 3:** Parameter estimation based on the wild bootstrap methods.

	Bootstrap mean	95% Bootstrap interval	
β_0_	-3.6584	-7.1043	-0.2112
β_1_	0.1826	0.1160	0.2494
β_2_	9.4934	7.4579	11.4626
R^2^	0.9891		

Table 4 reports predictions for Australia and U.K.; these datapoints are possible “outliers” when we do not take country-level variability into account. However, when we employ the mixed-effect regression, the predicted approval ratings of 92.54% and 97.92% for non-supporters and supporters in Australia are very close to the observed approval ratings of 93% and 98%, respectively. This is similar for the U.K., whose predicted approval ratings of 37.59% and 70.69% are for non-supporters and supporters, while the observations are 37% and 70%, respectively. However, the situation is very different from the U.S.

**Table 4:** Predictions based on the wild bootstrap methods.

Country	Y_i_ Observed	Support	$\hat{Y_{i}}$ Predicted	95% Bootstrap prediction interval	
Australia	93	0	92.5444	91.8812	93.1111
	98	1	97.9194	97.7408	98.0794
UK	37	0	37.5935	36.1849	39.0774
	70	1	70.6888	69.3976	72.0397
U.S.	29	0	35.2810	27.8156	42.9564
	76	1	71.7888	64.7408	77.8908

We find that the most significant residuals appear for the responses of the U.S., in which the predicted approval ratings are 35.28% and 71.79% for non-supporters and supporters while the observed approval ratings are 29% and 76%, correspondingly. The observed approval rating for governing party non-supporters of the U.S. is lower than the predicted value. This study reveals partisan polarization in the U.S. on COVID-19 policies, which agrees with [5, 19]. [7] display that affective polarization influences people’s evaluations of the U.S. government’s response to the COVID-19 pandemic. Opposing partisans are most likely to express negative sentiments about the governing party. Non-supporters of incumbents in the U.S. give “strict” ratings to their governing party, but “generous” ratings in favorable terms come from supporters to their fellow partisans.

Studies suggest that the U.S. COVID-19 response at that time was affected by its political leader [7, 20]. The COVID-19 pandemic brought severe threats to the U.S. labor market such as an increase in the unemployment rate, circuit breakers halting the U.S. stock market’s fall, and shocks to the economy and public health [21–23]. These events led to a more recent polarization observed in the U.S. The largest partisan gap in the assessments of the pandemic in this study is from the U.S., as the pandemic exacerbated partisan divisions in the country. The two parties disagreed on public health strategies ranging from mask-wearing to contact tracing [11]. Therefore, we observe a wider gap of approval ratings in the U.S. between Republicans’ and Democrats’ views of incumbent performance.

## 5. Conclusions

This study investigates the association and relationship of political partisanship and public opinion concerning governments’ responses to the COVID-19 pandemic via an international perspective. The mixed-effect regression allows for the relationship between approval ratings during the pandemic and two explanatory variables to vary across the country.

The approval ratings of citizens regarding their governments’ responses to the pandemic in September 2020 are based on the attitudes of supporting governing parties and the approval ratings toward such parties in 2019, or the pre-pandemic period. The most important factors in public opinions of a government’s performance in dealing with COVID-19 are partisanship and pre-pandemic approval ratings from the views between governing coalition supporters and opponents. This study deals with cross-sectional data, and if we can collect more datapoints in the near future, then we will be able to monitor the dynamics of political support even further.

As a final remark, the COVID-19 vaccination policy and its implementation became a primary task for governments in 2021. Many challenges still lie ahead for them. A government’s COVID-19 vaccination policy, including its vaccine acquisition, distribution plans, and prioritization approaches, can be additional potential public opinion factors regarding satisfaction relating to governments’ response to the COVID-19 pandemic in 2021.
